# Effectiveness of diode laser trans-scleral cyclophotocoagulation in patients following silicone oil-induced ocular hypertension in Chinese eyes

**DOI:** 10.4103/0301-4738.73730

**Published:** 2011

**Authors:** Rita Gangwani, David T L Liu, Nathan Congdon, Philip T H Lam, Vincent Y W Lee, Nancy S Y Yuen, Dennis S C Lam

**Affiliations:** Department of Ophthalmology and Visual Sciences, The Chinese University of Hong Kong, Prince of Wales Hospital, Hong Kong Special Administrative Region, China

**Keywords:** Ocular hypertension, pars plana vitrectomy, silicone oil, trans-scleral cyclophotocoagulation

## Abstract

We evaluated the effectiveness of diode laser trans-scleral cyclophotocoagulation (TSCPC) on intraocular pressure (IOP) in nine patients having raised IOP following use of silicone oil (SO) for retinal detachment (RD) surgery in a retrospective observational case series. Diode laser TSCPC was applied at a power setting of 1.75 to 2.5 watts, for two sec with a maximum of 30 applications. The patients were followed up for 40 to 312 weeks. The mean pre-laser IOP was 32.06 mm Hg (SD 7.32). The mean post-laser IOP at one month, three months and six months was 17.89 mm Hg (SD 8.23), 21.89 mm Hg (SD 8.16) and 21.67 mm Hg (SD 7.55) respectively. The final IOP (at the last follow-up) was 19.56 mm Hg (SD 7.85) (P=0.021). Seven of them had undergone SO removal. In our observation, effectiveness of TSCPC in long-term control of SO-induced ocular hypertension was limited as compared to short-term control of IOP.

Silicone oil (SO) is an indispensable tool for management of complicated vitreoretinal (VR) problems like tractional retinal detachment (RD) or proliferative vitreoretinopathy (PVR). However, its usage may be associated with raised intraocular pressure (IOP) or SO-induced glaucoma in 5.9-48% of cases.[1–3] A few reports have shown diode laser trans-scleral cyclophotocoagulation (TSCPC) as an effective tool in controlling IOP in SO-induced glaucoma.[4,5] We intended to look for short-term and long-term IOP control following diode laser TSCPC in Chinese eyes.

## Materials and Methods

Clinical records of nine patients who had undergone diode laser TSCPC for control of refractory SO-induced ocular hypertension (OHT) following RD surgery from January 2000 to December 2007 were reviewed. Ethics committee approval was obtained. Study parameters included primary VR diagnosis and surgery, chamber angle, anterior segment status, pre-laser IOP, post-laser IOP at one, three and six months, final IOP and number of laser sessions required.

All patients underwent pars plana vitrectomy (PPV) using three-port technique. Postoperative examinations were performed on Day 1, at one week, and then every two to three weeks depending on the retinal condition and IOP level.

Diode laser TSCPC was performed under local anesthesia using power of 1.75 to 2.5 watts, duration of two sec with a maximum of 30 applications over three quadrants, using a 600 micrometer laser delivery probe (G-probe); the probe was placed 1.5 mm posterior to the limbus. Following TSCPC, all patients were treated with prednisolone 1.0% eye drops four times a day. The pretreatment antiglaucomatous eye drops were continued. All patients were examined at one week after laser treatment initially. They were followed up for 40 to 312 weeks. Re-treatment was performed after six to eight weeks if the IOP was > 22 mm Hg. Seven patients had undergone SO removal and diode laser TSCPC was performed after SO removal.

We used Wilcoxon Signed Ranks test (SPSS Version 16.0) for statistical analysis.

## Results

The mean age of nine patients (four males and five females) was 57.22 ± 14.54 years (range 39-81 years). The mean pre-laser IOP was 32.06 ± 7.32 mm Hg (mean ± SD). The mean post-laser IOP at one month was 17.89 ± 8.23 mm Hg (*P*= 0.012), at three months was 21.89 ± 8.16 mm Hg (*P*= 0.03) and at six months was 21.67 ± 7.55 mm Hg (*P*= 0.038). The final IOP (at last follow-up) was 19.56 ± 7.85 mm Hg (*P*= 0.02).

Seven patients had a rise in IOP between two to eight weeks after SO infusion; two had IOP rise 20 weeks after SO infusion. None of them had raised IOP or glaucoma before SO infusion.

Two patients required single session of TSCPC; all others (78%) required ≥ 2 sessions of TSCPC for optimal IOP control (< 22 mm Hg) (mean - 2.34 sessions). Five patients underwent second session of TSCPC within six to eight weeks of first treatment while two patients required re-treatment more than 52 weeks (84 weeks) after first treatment. None of our patients had hypotony (IOP< 5 mm Hg) or any other complication.

We found that the IOP-lowering effect of TSCPC was much more at one to two months with subsequent rise in IOP at three months or beyond that; although final IOP remained controlled with repeated treatment and antiglaucoma eye drops. Six out of nine patients (66.6%) had final IOP of ≤ 21 mm Hg.

Central visual acuities were preserved or improved in eight patients and deteriorated (from 1/200 to hand movement) in one patient [Table 1]. Following TSCPC, use of oral carbonic anhydrase inhibitor (acetazolamide) was discontinued in three patients and topical antiglaucomatous medications were reduced in three patients.

**Table 1:** Pre-laser and post-laser details of patients undergoing trans-scleral cyclophotocoagulation

Pt. No.	Sex/Age	VR diagnosis	Time of SO removal*	No. of treatments	Pre-laser IOP	Post-laser (last f/u) IOP	f/u wks	Pre- laser med	Post -laser med
1	F/72	PDR+TRD	3	1	28.50	39	104	D+2	2
2	F/73	PDR+TRD	14	1	29	11	96	3	3
3	F/49	Myop.RRD	15	3	25	18	156	D+3	3
4	M/46	RRD	N/A	3	40	15	312	3	3
5	M/81	MH+RRD	15	2	20	18	40	D+3	3
6	M/39	Myop.RRD	4	5	40	22	312	4	3
7	F/56	RRD	20	2	29	16	92	D+3	D+3
8	F/52	PDR+TRD	9	2	37	23	92	3	2
9	M/47	PDR+TRD	N/A	2	38	14	104	3	0

Pt.: Patient, F: Female, M: Male, VR: Vitreoretinal, TRD: Tractional retinal detachment, PDR: Proliferative diabetic retinopathy, Myop.: Myopic; RRD = Rhegmatogenous retinal detachment, MH: Macular hole, Med.: Medication, D: Diamox (acetazolamide),

Time of SO removal* = time of SO removal in months after SO infusion

N/A: Not applicable, f/u: Follow-up, TSCPC: Trans-scleral cyclophotocoagulation

Pre-laser gonioscopic findings were documented in five patients (scattered peripheral anterior synechia in four patients, and open angle with presence of SO in angle in one). Six patients were pseudophakic (posterior chamber intraocular lens), two were aphakic and one was phakic.

## Discussion

The risk factors for SO-induced raised IOP are preexisting glaucoma, diabetes mellitus, aphakia, and surgery for recurrent rhegmatogenuos RD with PVR.[6,7]

IOP may return to normal levels following SO removal,[8] but in our experience, removal of SO alone was insufficient to control IOP which could be due to chronic inflammatory cellular infiltration of trabecular meshwork while SO is still in eye.[9]

The mechanism of SO-induced raised IOP remained controversial. SO may have chronic damaging effect to trabecular meshwork and reduced aqueous drainage.[9] The SO has tendency to migrate to the anterior chamber and trabecular meshwork causing synechial angle closure, rubeosis iridis, pupillary block and inflammation.[5,7] In some patients, it becomes extremely difficult to achieve equilibrium of aqueous dynamic in face of such a markedly reduced outflow facility even after multiple attempts of cycloablation. Ethnicity difference with regard to ocular biometry and predisposition to iridocorneal angles crowding may be another explanation of our data. In Chinese eyes anterior chambers are relatively shallow, with anterior position of ciliary body; there is a propensity for angle closure or even acute angle closure glaucoma.[10] This may be aggravated by presence of SO which has a physiochemical tendency for pushing the lens–iris plane forward with further narrowing of angles. But this factor needs to be evaluated further.

There are certain shortcomings concerning our study. The study was a retrospective one, with comparatively small number of patients. The patients belonged to a heterogeneous group and were not standardized in terms of treatment regimen. Two patients did not undergo SO removal because of retinal status. The number of laser applications was determined by treating surgeon and laser procedure was performed by more than one surgeon. Gonioscopic findings were documented in only five patients. There have been similar studies done before. We intended to look for short-term and long-term IOP control in Chinese eyes. Therefore in order to fully address this important management issue, a prospective study or a multicenter study in future with a larger group of patients and longer observation is warranted.

We conclude that although diode laser TSCPC was effective in reducing IOP, its effect was more encouraging in the short-term period requiring multiple sessions and adjuvant use of antiglaucoma medications for long-term IOP control. It might be of limited effectiveness as the only method for long-term IOP control.
